# Postprandial effects of calcium phosphate supplementation on plasma concentration-double-blind, placebo-controlled cross-over human study

**DOI:** 10.1186/1475-2891-12-30

**Published:** 2013-03-08

**Authors:** Ulrike Trautvetter, Michael Kiehntopf, Gerhard Jahreis

**Affiliations:** 1 Department of Nutritional Physiology, Institute of Nutrition, Friedrich Schiller University of Jena, Dornburger Straße 24, Jena, D-07743, Germany; 2 Institute of Clinical Chemistry and Laboratory Medicine, Jena University Hospital, Friedrich Schiller University Jena, Erlanger Allee 101, Jena, D-07747, Germany

**Keywords:** Calcium phosphate, Plasma calcium, Plasma phosphate, Human study

## Abstract

**Background:**

The aim of the present study was to examine the postprandial calcium and phosphate concentrations after supplementation with pentacalcium hydroxy-triphosphate (CaP).

**Methods:**

Ten men participated in this double-blind, placebo-controlled, cross-over study. The participants were divided into two groups. One group consumed bread enriched with CaP (plus 1 g calcium/d) and the other group a placebo product for three weeks. After a two week wash-out, the intervention was switched between the groups for another three weeks. Blood samples were drawn at the beginning (single administration) and at the end (repeated administration) of the intervention periods at 0, 30, 60, 120, 180 and 240 min. Between 0 and 30 min, a test meal, with or without CaP was consumed. The plasma concentrations of calcium and phosphate were examined. One participant dropped out due to personal reasons.

**Results:**

CaP supplementation resulted in a significantly higher plasma calcium concentration after 240 min compared to placebo. After repeated CaP administration, the AUC for the increment in plasma calcium concentration was significantly higher compared to placebo.

After single and repeated CaP supplementation, plasma phosphate concentration significantly decreased after 30, 60, 120 and 180 min compared to 0 min. The placebo administration resulted in significant decreases after 30, 60 and 120 min compared to 0 min.

**Conclusion:**

Our results show that CaP contributes to an adequate calcium supply, but without increasing the plasma concentration of phosphate.

**Trial registration:**

http://www.clinicaltrials.gov; NCT01296997

## Background

Hyperphosphatemia is recognized as a risk factor for mortality in chronic kidney disease [1]. In addition, serum phosphate concentration within the upper limits of normal is associated with a greater prevalence of vascular and valvular calcification in patients with moderate chronic kidney disease [2]. In the last years, our research group performed human studies involving calcium phosphate supplementation [3-5]. Most studies with calcium phosphate focus on the beneficial effects relating to intestinal metabolism, e.g. bile acid metabolism, fatty acid excretion, and modulation of the microbiota [5-8]. This is because amorphous calcium phosphate is formed in the human gut and, moreover, is able to precipitate intestinal substances, such as bile or fatty acids [6,7,9]. However, calcium phosphate is poorly absorbed in the gut. Evidence comes from studies showing unchanged fasting plasma concentrations of calcium and phosphate after calcium phosphate supplementation [4]. Nevertheless, measuring fasting concentrations is not a convincing method to examine the influence of calcium phosphate supplementation on calcium and phosphate status. Furthermore, Heaney *et al*. showed that solubility of a calcium supplement has very little influence on its absorbability and that absorption of calcium from food sources is determined mainly by other food components [10]. In addition, it is necessary to test every calcium product for absorbability [11]. Therefore, in this human study, we examined the postprandial calcium and phosphate concentrations after calcium phosphate supplementation of both a single dose and after three weeks.

## Methods

### Supplement

For purposes of supplementation, we used pentacalcium hydroxy-triphosphate (Ca_5_(PO_4_)_3_OH; cfb; Budenheim Germany; CaP) in this study. CaP was incorporated in whole wheat bread to achieve an additional calcium intake of 1 g/d (0.5 g phosphorus/d). Participants consumed approximately 135 g of this bread daily. Placebo bread was prepared in exactly the same way, but without the CaP supplement.

### Subjects

**Figure 1:**
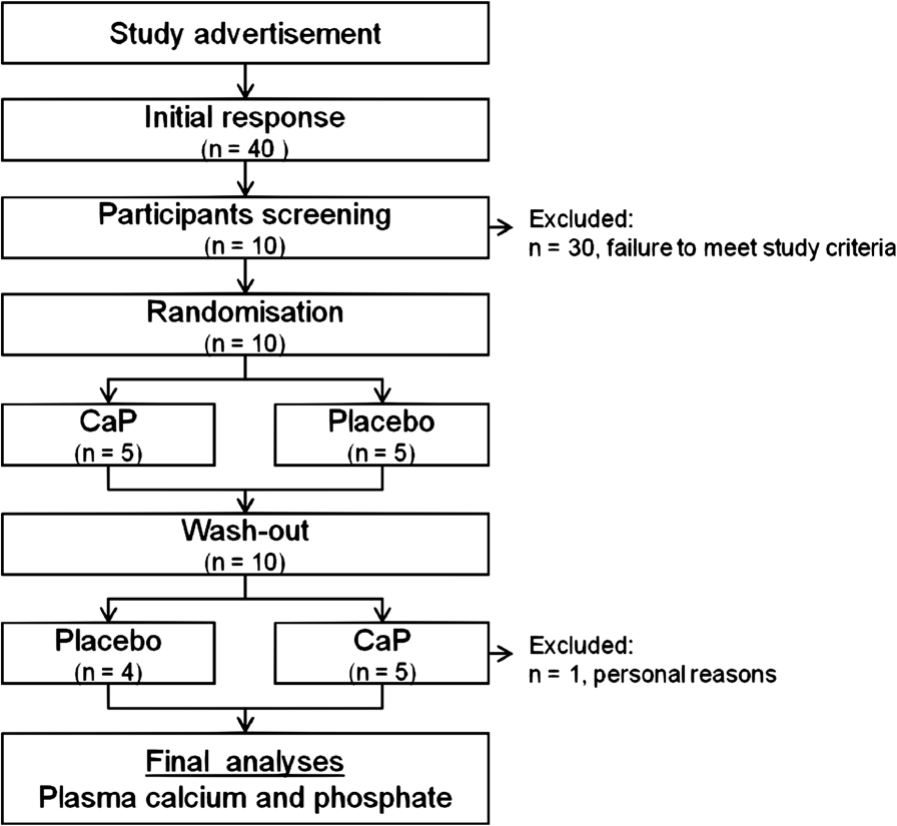
**Study flowchart.** CaP: pentacalcium hydroxy-triphosphate.

The study was conducted between July and September 2010 in the Institute of Nutrition, Department of Nutritional Physiology at the Friedrich Schiller University Jena. Ten omnivorous men participated in this double-blind, placebo-controlled, cross-over study. Eligibility criteria for participants included age between 20 and 35 years and good physical health. A further criterion was that participants remain at the blood withdrawal centre for at least 5 hours between 7.30 am till 12.30 pm. Volunteers were provided with detailed information regarding purpose, course, and possible risks involved in the study. All participants gave their written informed consent. The study protocol was approved by the Ethical Committee of the Friedrich Schiller University, Jena (No.:2833-05/10). Of the initial ten volunteers, one participant dropped out due to personal reasons. The main study outcomes comprised blood concentrations of calcium and phosphate (Figure 1).

### Study design

**Figure 2:**
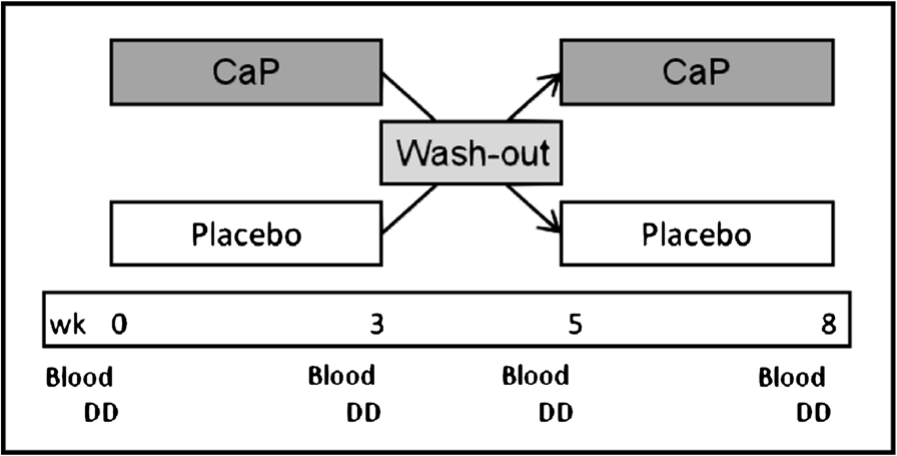
**Design of the double-blind, placebo-controlled cross-over study.** wk: week; CaP: pentacalcium hydroxy-triphosphate; blood: blood sampling at time points 0, 30, 60, 120, 180 and 240 minutes; between 0 and 30 minutes, the participants consumed a test meal with or without CaP; DD: defined diet for three days before blood sampling.

Participants were divided into two groups. For a period of three weeks, one group consumed bread containing CaP whereas the other group consumed the placebo product. This was followed by a two-week wash-out phase. Thereafter, the intervention changed between the two groups for a further three weeks. Thus, the study design allowed that every participant was his own control. The intervention periods are divided in two parts. At the beginning the participants ate the test bread with or without CaP onetime and then blood was taken (single administration). Afterwards the participants consumed the test bread three weeks daily and then blood was taken again (repeated administration). Consequently, the study involved four types of administrations: single CaP administration, single placebo administration, repeated CaP administration and repeated placebo administration. In addition, participants consumed a defined diet for three days before each blood sample was taken (Figure 2).

The defined diet containing the complete food supply for three days was prepared and pre-weighed in the study centre. The subjects were instructed to consume no other foods than provided. Any food residues were weighed and food intake was calculated.

On the day of blood withdrawal, participants came fasting to the Institute of Transfusion Medicine of the Jena University Hospital. Blood samples were drawn after 0, 30, 60, 120, 180 and 240 minutes, immediately cooled, and transported to the study centre.

Between time point 0 and 30 minutes, the participants consumed a test meal. The test meal consisted of bread with or without CaP (according to the intervention), 20 g butter, 25 g ham, 15 g sweet hazelnut spread and a banana. During the 240 min the participants were allowed to drink water *ad libitum.*

### Sample preparation

Samples of each food component of the defined diet were frozen and stored at −20°C until analysis. Blood was collected in lithium heparin tubes. Plasma was obtained by centrifugation at 2500 x g for 15 minutes at 20°C. Aliquots were frozen at −80°C until analysis.

### Food analysis

The intake of energy, fat, proteins, and carbohydrates was verified using the Prodi® 5.4 software (Nutri-Science GmbH, Freiburg, Germany). For intake of minerals, the respective contents in the provided foods were analysed instead of using the calculation software. Mineral contents of all food samples were determined employing the iCAP 6000 ICP Spectrometer (Thermo Scientific, Waltham, USA). Before analysis, the samples were ashed at 525°C. The ash was dissolved in HCl (25%) and diluted with distilled water.

### Analysis of calcium and phosphate

Calcium and phosphate in plasma were quantified using the autoanalyser ARCHITECT C16000 (Abbott, Illinois, USA) according to the manufacturer’s recommendations.

### Calculations and statistical methods

Samples from each participant were coded to protect volunteer identity and to mask treatment groups during the analysis. The areas under the curves (AUC) from 0 to 240 min were calculated using the trapezoidal method. The calculation based on the increment in plasma calcium and phosphate concentrations.

All values in the text and tables were expressed as mean ± standard deviation. For reasons of clarity and comprehensibility, values in figures were expressed only as means. Data analysis was performed using the statistical software package PASW Statistics 18 (SPSS Inc., Chicago, USA). Differences were considered significant at p ≤ 0.05. The effect of time was tested only to baseline using paired Student’s t-test. The effect of supplementation was tested with paired Student’s t-test. The sample size for calcium and phosphate was n = 9.

## Results

Nine subjects completed the four blood samples and test meals. The baseline characteristics and the nutrient intake of the test meal and defined diet are presented in Tables 1 and 2**.**

**Table 1:** Baseline characteristics of participants who completed the study

	**Subject (n= 9)**
Age [y]	27 ± 4
Height [cm]	178 ± 5
Weight [kg]	73.2 ± 7.5
BMI [kg/m^2^]	23.1 ± 2.3

**Table 2:** Mean nutrient composition of the test meal and the defined diet

	**Test meal (per meal)**		**Defined (per day)**	
Energy [MJ]	2,6 ± 0,1		10,4 ± 1,4			
Carbohydrates [g]	82,1 ± 3,4		292,9 ± 41,2			
Fat [g]	23,5 ± 0,1		101,6 ± 19,1			
Protein [g]	16,7 ± 0,3		85,3 ± 6,3			
	CaP	Placebo	CaP	Placebo		
Ca [mg]	1160 ± 1	55 ± 1	2019 ± 75,2	926 ± 57		
from CaP [mg]	1104	0	1104	0		
P [mg]	868 ± 4	346 ± 5	2050 ± 127	1542 ± 104		
from CaP [mg]	519	0	519	0		

N = 9; data are expressed as mean ± standard deviation; CaP pentacalcium hydroxy-triphosphate, Ca calcium, *P* phosphorus.

The calcium and phosphorous concentrations of the CaP bread was 1129 mg/135 g bread and 749 mg/135 g bread, respectively. The placebo bread contained 26 mg calcium/135 g bread and 229 mg phosphorus/135 g bread, respectively.

**Figure 3:**
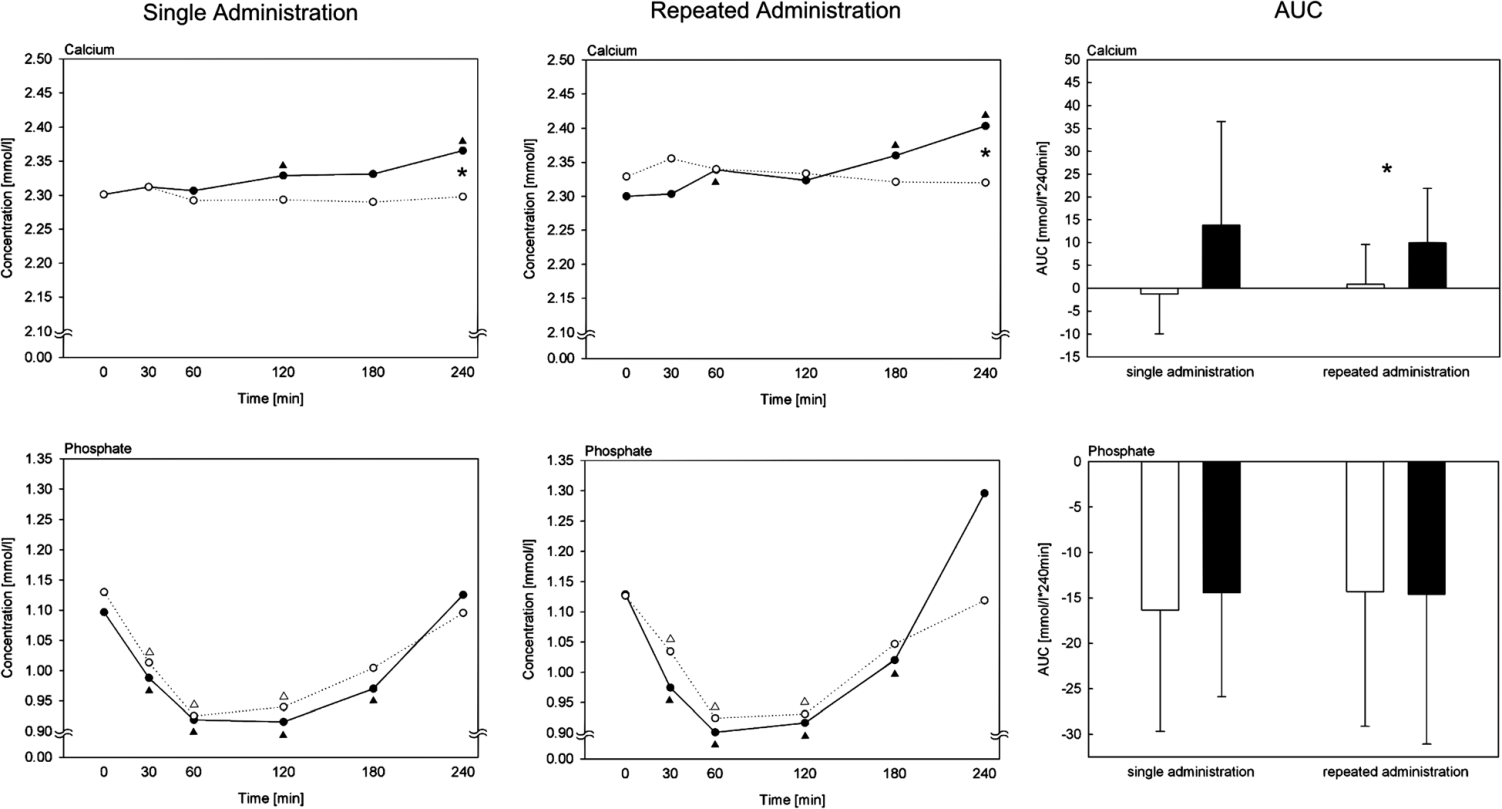
**Mean plasma calcium and phosphate concentrations and area under the curve from 0 to 240 min for the increment in plasma calcium and phosphate after single and repeated administration of CaP.** N = 9; data are expressed as means, areas under the curve are expressed as mean ± standard deviation;▲ significant differences to 0 min after CaP supplementation (p ≤ 0.05); ○ placebo; ● CaP supplementation;∆ significant differences to 0 min after placebo administration (p ≤ 0.05); * significant differences between CaP and placebo administration (p ≤ 0.05); the effects of time and of supplementation were tested with paired Student’s t-test; CaP: pentacalcium hydroxy-triphosphate; AUC: area under the curve.

Following single administration with CaP, calcium concentration increased significantly after 120 (p = 0.043) and 240 min (p ≤ 0.001) compared to 0 min. The three week administration of CaP led to an increase of calcium concentration after 60 (p = 0.026), 180 (p = 0.011) and 240 (p = 0.001) min compared to 0 min (Figure 3). Both the single and the repeated administration of CaP resulted in a significantly higher calcium concentration after 240 min (p = 0.005, p = 0.006) compared to placebo. After repeated administration, the AUC for the increment in calcium concentration was significantly higher after CaP administration compared to placebo (p = 0.007).

After single and repeated CaP supplementation, the phosphate concentration significantly decreased after 30 (single: p ≤ 0.001, repeated: p = 0.002), 60 (single: p ≤ 0.001; repeated: p ≤ 0.001), 120 (single: p = 0.006, repeated: p = 0.006) and 180 (single: p = 0.043, repeated: p = 0.041) min compared to 0 min. The placebo administration resulted in similar significant decreases after 30 (single: p ≤ 0.001; repeated: p = 0.001), 60 (single: p ≤ 0.001; repeated: p ≤ 0.001) and 120 (single: p = 0.007; repeated: p = 0.011) min compared to 0 min.

Following single CaP and placebo administration, the calcium-phosphorus product significantly decreased compared to 0 (CaP: 2.5 mmol^2^/l^2^; placebo: 2.6 mmol^2^/l^2^) after 30 (CaP: 2.3 mmol^2^/l^2^, p ≤ 0.001; placebo: 2.3 mmol^2^/l^2^, p = 0.003), 60 (CaP: 2.1 mmol^2^/l^2^, p ≤ 0.001; placebo: 2.1 mmol^2^/l^2^, p ≤ 0.001), 120 (CaP: 2.1 mmol^2^/l^2^, p = 0.006; placebo: 2.2 mmol^2^/l^2^, p = 0.008) and 180 (CaP: 2.3 mmol^2^/l^2^, p = 0.049; placebo: 2.3 mmol^2^/l^2^, p = 0.031) min, respectively. After repeated CaP and placebo administration, the calcium-phosphorus product significantly decreased after 30 (CaP: 2.4 mmol^2^/l^2^, p = 0.001; placebo: 2.4 mmol^2^/l^2^, p = 0.001), 60 (CaP: 2.1 mmol^2^/l^2^, p = 0.001; placebo: 2.2 mmol^2^/l^2^, p ≤ 0.001) and 120 (CaP: 2.1 mmol^2^/l^2^, p = 0.011; placebo: 2.2 mmol^2^/l^2^, p = 0.012) min compared to 0 min (CaP: 2.6 mmol^2^/l^2^, placebo: 2.6 mmol^2^/l^2^). At 0 min, the calcium-phosphorus product was significantly higher after CaP administration compared to placebo administration (p = 0.031).

## Discussion

Calcium has a tightly regulated homeostasis rendering it unsuitable for comparing fasting calcium concentrations for the purpose of determining the effect of supplementation on calcium status. Although, there are some studies in the literature in which the calcium concentration was determined following a short time interval after calcium intake [12-16]. For instance, Heaney *et al.* compared the bioavailability of calcium from two fortification systems. A combination of tricalcium phosphate and calcium lactate (500 mg calcium, orange juice) led to an increase in serum calcium of almost 0.35 mg/dl (0.09 mmol/l) after two hours [14]. In the study of Green *et al.*, the change in the calcium concentration after tricalcium phosphate (1200 mg calcium, powder mixed in water) was 0.1 mmol/l [12]. In present study, plasma calcium concentration rose by approximately 0.06 mmol/l in four hours after single administration and by about 0.1 mmol/l after repeated administration. The significantly higher calcium concentration at 240 min after single and repeated CaP administration and the significantly higher AUC for the increment in calcium concentration after repeated CaP administration compared to placebo suggest that part of the calcium from the CaP supplement was absorbed. Supplementation with CaP led to an increase in blood calcium concentration, which is comparable to other studies.

Because hyperphosphatemia is linked with vascular calcification, cardiovascular mortality, and progression of chronic kidney disease, phosphate intake is an aspect that is controversially discussed [17-19]. In the present study, there was no difference in the plasma phosphate concentration between CaP and placebo after 240 min. In another study by Reginster *et al.* comprising 10 male subjects, the serum phosphorus concentration did not change after supplementation with tricalcium phosphate (1000 mg Ca) within a time frame of 360 min [20]. In contrast, both Yang *et al.* and Shires *et al.* showed an increase in phosphorus concentration after an intake of 1200 mg calcium [21,22].

Interestingly, after all administrations (single CaP and placebo administration, repeated CaP and placebo administrations), phosphate concentration significantly decreased at first, followed by a rise to basal concentration (Figure 2). Karp *et al.* showed similar results for plasma phosphate concentration after supplementation with different calcium supplements and potassium citrate [23]. Because of a decrease in bone resorption, the authors assumed a decrease in the release of phosphate from bone for potassium citrate only [23]. Moreover, hypophosphatemia can be a result of phosphate shifts from the blood into the cells, such as after ingestion of glucose, fructose, and feeding following starvation (phosphorylation processes) [24]. A glucose load can induce a transient reduction in serum phosphate levels [24], probably due to secretion of gastrointestinal hormones and subsequent stimulation of calcitonin [25]. Calcitonin induces an inhibition of osteoclasts and a reduction in the release of phosphorus from bone into blood. However, the association between gastrointestinal hormones and calcitonin has only been shown in rodents and not in humans [26].

Alternatively, the decreasing concentration of phosphate in the present study can also be explained by a dilution effect as participants were allowed to drink water *ad libitum* after the test meal. Between time points 60–120 min, the phosphate concentration increased again to baseline levels indicating that intestinal phosphate absorption took place within this time frame. Indeed, in the study by Karp *et al*., the participants were also allowed to drink water *ad libitum*[23]. In contrast, studies in which liquid intake were restricted showed either an immediate increase or a constant phosphate concentration [20-22].

Hence, the present results indicate that the increased phosphate intake due to the CaP supplementation, did probably not lead to an increased phosphate absorption in the gut. This conclusion is supported by comparably postprandial phosphate concentrations and the similar AUC for the increment in phosphate concentration after CaP and placebo administrations. In order to confirm these results, isotopic tracer studies are indicated. However, the results show that phosphate from this CaP supplement did not lead to an increase in plasma phosphate concentration.

## Conclusion

In conclusion, our results show that CaP contributes to an adequate calcium supply, but without increasing the plasma concentration of phosphate. Indeed, the major part of the phosphate from the CaP supplement is precipitated as amorphous calcium phosphate in the human gut, an aspect that has been shown in several human studies [3,5,27,28].

## Abbreviations

CaP: Pentacalcium hydroxy-triphosphate; AUC: Area under the curve.

## Competing interests

The authors declare that they have no competing interests.

## Authors’ contribution

UT conducted research, wrote the manuscript and performed statistical analysis; UT and MK analysed data; UT and GJ designed research and had primary responsibility for final content. All authors read and approved the final manuscript.
